# Mendelian randomisation analysis of clustered causal effects of body mass on cardiometabolic biomarkers

**DOI:** 10.1186/s12859-018-2178-2

**Published:** 2018-07-09

**Authors:** Susana Conde, Xiaoguang Xu, Hui Guo, Markus Perola, Teresa Fazia, Luisa Bernardinelli, Carlo Berzuini

**Affiliations:** 1 Centre for Biostatistics, University of Manchester, University Place, Oxford Road, Manchester, M13 9PL UK; 2 National Institute for Health and Welfare (THL), P.O. Box 30 (Mannerheimintie 166), Helsinki, FI-00271 Finland; 3 Department of Brain and Behavioural Sciences, University of Pavia, Via Bassi 21, Pavia, 27100 Italy

**Keywords:** Cardiovascular disease, Causal inference, Egger regression, Metabolomics, Multiple instrumental variables, Weighted median estimator

## Abstract

**Background:**

Recent advances in data analysis methods based on principles of Mendelian Randomisation, such as Egger regression and the weighted median estimator, add to the researcher’s ability to infer cause-effect links from observational data. Now is the time to gauge the potential of these methods within specific areas of biomedical research. In this paper, we choose a study in metabolomics as an illustrative testbed. We apply Mendelian Randomisation methods in the analysis of data from the DILGOM (Dietary, Lifestyle and Genetic determinants of Obesity and Metabolic syndrome) study, in the context of an effort to identify molecular pathways of cardiovascular disease. In particular, our illustrative analysis addresses the question whether body mass, as measured by body mass index (BMI), exerts a causal effect on the concentrations of a collection of 137 cardiometabolic markers with different degrees of atherogenic power, such as the (highly atherogenic) lipoprotein metabolites with very low density (VLDLs) and the (protective) high density lipoprotein metabolites.

**Results:**

We found strongest evidence of a positive BMI effect (that is, evidence that an increase in BMI causes an increase in the metabolite concentration) on those metabolites known to represent strong risk factors for coronary artery disease, such as the VLDLs, and evidence of a negative effect on protective biomarkers.

**Conclusions:**

The methods discussed represent a useful scientific tool, although they assume the validity of conditions that are (at best) only partially verifiable. This paper provides a rigorous account of such conditions. The results of our analysis provide a proof-of-concept illustration of the potential usefulness of Mendelian Randomisation in genomic biobank studies aiming to dissect the molecular causes of disease, and to identify candidate pharmacological targets.

**Electronic supplementary material:**

The online version of this article (10.1186/s12859-018-2178-2) contains supplementary material, which is available to authorized users.

## Background

Ideally, the causal effect [1, 2] of a phenotype or exposure (*X*) on an outcome (*Y*) should be assessed by setting up an experiment, where we have control on the value of *X* in each experimental unit. Unfortunately, the experimental approach is not universally applicable: in a wide range of research fields and situations we shall be forced to assess a causal effect of interest via an observational study, that is, a study where we have no control on the values of *X*, and where all we can do is to passively observe the values of that variable in each sample unit. Sometimes *X* represents a variable that is inherently uncontrollable in humans in an experimental setting as is the case with, say, body mass and smoking habit.

Unfortunately, the observational assessment of a causal effect is highly vulnerable to biases. No matter how immaculate is our study design, we shall generally be unable to eliminate sources of bias like confounding and reverse causation between *X* and *Y*. We tackle this problem in a study of the possible causal effect of body mass (*X*) on the concentration of each of a collection of 137 cardio-metabolites. Our main analysis tool are methods of Mendelian randomization (MR) [3–5], in combination with techniques of multivariate data analysis. The basic idea behind these methods is that for *X* to be a causal influence on *Y*, we, under certain assumptions, expect a genetic variant *Z* that modulates *X* to likewise affect *Y*. The mathematical methods we illustrate in this paper use information about *Z* to assess the causal effect of *X* on *Y* without the need to conduct an experiment. In this case, we say that *Z* acts as an *instrument* in the assessment of the causal effect of *X* on *Y*. The term “instrument” echoes the name of another important class of methods for assessing causal effects, known as instrumental variable [6] analysis (IV analysis). It is common to use the term “Mendelian randomisation” rather than “IV analysis” when, as in our case, the instrumental variables are genetic variants associated with *X* (in our case, with BMI). The instrumental use of genetic variables is very fruitful in the era of genomewide genetic association and expression studies, which provide us with a wealth of information about relationships between varuants and phenotypes. In fact, MR provides us with a coherent framework for transforming the growing body of genome-wide association knowledge into causal knowledge.

MR methods may play an important role in pharmacological and medical research [7]. They have been used to predict the outcome of clinical trials and to test the causal hypothesis that motivates them [8]. Moreover, MR analysis of biobank data can be used to validate causal hypotheses of therapeutic and pharmacological relevance, e.g. the putative causal effect of a protein on a disease.

Early implementations of MR used one or few instrumental variants, under the untestable assumption that these variants are not pleiotropic, i.e., that they affect the outcome only through the changes they induce in the exposure. Later developments have produced methods that use multiple instruments, while allowing some (unspecified) subset of them to affect the outcome directly. Among these are the Egger regression (ER) and the weighted median estimator (WME) [9] methods, which we use in this paper.

Our illustrative study takes the putative causal phenotype, *X*, to represent body mass, as measured by the body mass index (BMI), and considers a collection of outcomes representing the concentrations of a large array of cardiometabolic markers. The markers can be subdivided into biologically meaningful clusters. One of these is a cluster of very low density lipoprotein (VLDL) metabolites, known to be highly atherogenic (in the sense that they tend to induce atherosclerotic plaques). Other clusters comprise correlated sets of moderately atherogenic or neutral metabolites. In this paper, we use MR methods to assess the causal effect of BMI on each of the cardiometabolites in the study, and analyse the variability of the causal effect within and across the clusters. Our MR analyses suggest that BMI exerts a significant causal effect on each of a large subset of the studied metabolites, and that the effects of BMI on the cardiometabolites belonging to the same cluster tend to be homogeneous and with the same sign. In particular, our analysis suggests that BMI tends to increase the concentrations of those markers that are known to represent strong risk factors for coronary artery disease.

## Methods

### Study motivation and data

Excess of body fat, also called adiposity, is a growing threat to public health, despite the fact that it can be prevented or reversed by eating less and exercising more. A popular measure of body fat, BMI, is defined as the person’s weight (kilograms) divided by the square of their height (meters^2^). Although this measure fails to capture variation in the way the mass of fat is distributed in the body, it is regarded as a useful summary for large epidemiological studies. Individuals with higher BMI suffer from a higher risk of developing such cardiometabolic diseases as heart failure and stroke. There is evidence (see, for example, [10]) that the increased risk of disease and death associated with excess adiposity is partly attributable to abnormalities in the way individuals with high adiposity metabolize carbohydrates and fats, these abnormalities being responsible for a higher concentration of blood sugar and cholesterol levels. This motivates interest in the changes induced by BMI in an individual’s systemic metabolic profile – so far a largely unexplored theme. Würtz and colleagues [11] point out that if there is a causal effect of adiposity on cardiometabolic risk factor levels, it might be possible to prevent the progression towards cardiometabolic diseases by weight loss.

A number of authors tackle the problem by calculating the association between BMI and each metabolite of interest. However, these associations are of limited usefulness. They will generally differ from the causal effects of interest, due to the relationship between BMI and each metabolite concentration being generally confounded by a constellation of unobserved biological agents. Luckily, we are able to estimate those causal effects via MR. This idea is exploited by Würtz and colleagues [11], who perform a MR analysis where BMI acts as exposure (*X*), cardiometabolic markers act as outcomes and genetic variants associated with BMI act as instruments. One limitation of Würtz’s MR analysis is that it was performed by using a two-stage least squares approach, which assumes validity of the questionable “no-pleiotropy” condition (see Condition (*c*) in the next subsection).

In the remaining part of this paper, we perform a MR analysis along lines similar to Würtz and colleagues [11], this time by using up-to-date MR methods, notably ER and WME, which will dissolve the fear of biases due to unaccounted-for pleiotropy.

Our analysis is based on data from the Dietary, Lifestyle and Genetic determinants of Obesity and Metabolic Syndrome (DILGOM) study [12]. This study contains observational measures for 137 metabolite concentrations in *n*=520 subjects from a Finnish population cohort plus various phenotypic features, including BMI, age, sex, ∼ 35,500 gene expressions and 38 millions of single nucleotide polymorphisms (SNPs) measured in *n*=688 subjects who include the 520.

Cardiovascular disease risk is thought to be causally affected by the concentrations of such metabolites as low density lipoprotein (LDL) and high density lipoprotein (HDL). The DILGOM Study exploits the possibility of measuring these lipoproteins in the plasma in terms of particle numbers, sizes and concentrations, in contrast to the traditional measures restricted to concentrations. Thus, our data files contains information about such metabolites as small, medium and large-sized lipoprotein particles, for a more informative analysis. The small LDL particles are considered more atherogenic (generators of atherosclerosis) than the large LDL particles.

From a substantive point of view, our aim is to use instrumental information extracted from a genomewide list of SNPs to investigate the existence of causal relationships between BMI and the concentrations of cardiometabolic markers, and to assess whether a high BMI tends to increase the concentration of those metabolites that are known to be highly atherogenic, that is, responsible for an increased risk of atherosclerotic plaques and life-threatening coronary artery disease.

### Mendelian randomisation assumptions

**Fig. 1:**
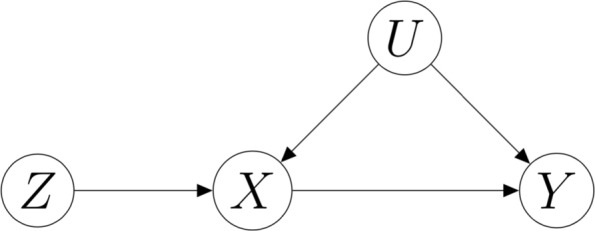
Conditional independence graph representation for a class of Mendelian randomisation problems that does not violate Conditions (**a**), (**b**) and (**c**) in the main text. Here *Z* represents the instrumental variable(s), the symbol *X* represents an (intermediate) phenotype or exposure, *Y* represents the outcome, and *U* a set of imperfectly observed confounders of the relationship between exposure and outcome

It is sometimes possible, and in such a case helpful, to discuss the assumptions at the basis of MR with the aid of the conditional independence graph in Fig. 1, where the nodes represents random variables in the problem, and the arrows may, or may not, represent causality. The graph may be interpreted to represent the class of all joint distributions over the graph variables that satisfy all the conditional independence relationships that the graph imposes between the variables. These relationships can be read off by using Pearl’s *d*-separation criterion [13], which we omit from this discussion.

In our study the exposure, denoted by *X*, is the natural logarithm of BMI, whereas the outcome, *Y*, is the natural logarithm of the concentration of the generic cardio-metabolite. Symbol *Z* represents the generic instrumental SNP, with value in (0,1,2) according as the SNP allele is the homozygous major “AA”, the heterozygous “Aa”, or the homozygous minor “aa”, respectively.

Figure 1 shows a graph where one node, *U*, represents the set of imperfectly observed (perfectly known to Nature, but not to us) confounders, which are responsible for the correlation between *X* and *Y* not being entirely attributable to causation [2]. It is sometimes assumed in MR that *Z* (in our study the generic instrumental variant) satisfies the following conditional independence relationships:

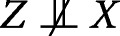
,
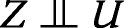
,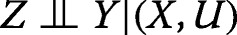,

where the phrase “
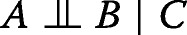
” stands for “*A* is conditionally independent of *B* given *C*” [14], and 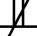 for “not independent of”. Condition (*a*) may be interpreted as requiring that each instrumental SNP be associated with the exposure *X*, whereas Condition (*b*) requires the instrument to be independent of the confounders, and Condition (*c*) excludes pleiotropic associations, that is, any instrument-outcome association (or causal effect of the instrument on the outcome) that is not entirely mediated by the exposure. None of the three conditions is violated by the graph in Fig. 1. In particular, Condition (*b*) follows from the absence of an unblocked (in the sense of *d*-separation) path between nodes *X* and *U* in the graph. Condition (*c*) follows from the fact that the node pair (*X*,*U*) blocks all paths between *Z* and *Y* in the graph.

Presence of an arrow from node *Z* to node *X*, in the graph, should not prevent the reader from interpreting Condition (*a*) as compatible with instruments associated with the exposure in a non-causal way. In most situations we shall not know the variants that are causal for *X*. Many instruments, typically single nucleotide polymorphisms (SNPs), will be associated with *X*, but simply because they are in linkage disequilibrium (LD) with an unobserved causal variant. Luckily, the methods we use are compatible with this type of non-causality, provided the instrumental variants are independent of each other.

Pleiotropic associations incompatible with (*c*) (but compatible with (*b*)) are often represented in the graph by a *Z*→*Y* arrow (missing in Fig. 1). They arise, for example, when an instrumental SNP is associated with both *X* and an unknown variant that exerts a causal effect on *Y* independent of *X*. Presence of pleiotropy that violates (*c*) is biologically plausible but statistically untestable. Luckily, the methods used in this paper are robust to violations of this condition, provided (*a*) and (*b*) are satisfied. This means that they allow some of the instruments, *which we are not asked to identify*, to have an association with *Y* that is not mediated by either *X* or *U*. The flip side of this is that we need to introduce a further condition requiring that the pleiotropic effects be *un*correlated with the associations of the instruments with the exposure. This is called the Instrument Strength Independent of Direct Effect (InSIDE) condition, and is untestable.

If the association of an instrumental SNP with *X* (BMI in our study) is very significant, it is said to be a “strong instrument”. If it is scarcely significant, we say that the instrument is weak. Presence of weak instruments, especially in conjunction with a small sample size, may induce bias in the estimate of the causal effect.

Because Conditions (*b*) and InSIDE are not testable, and cannot be eschewed, the conclusions from an MR analysis will always have to be interpreted with caution.

In any specific application, an important task will be to search the genome for genetic variants to act as instruments in the MR analysis. In our study, these were selected genomewide from among all the SNPs associated with BMI, in compliance with Condition (*a*), under the assumption that they also comply with (*b*).

We assume that dependence of *X* and *Y* on their direct influences in the graph is governed by a linear normal additive regression, where each SNP, *Z*, takes values (0,1,2) on an interval scale, so that the expected increment in either X or Y due to an additional occurrence of the minor allele is represented in the model by a single unknown parameter. Note that we are assuming *X* and *U* not to interact in their effects on *Y*, and *Z* and *U* not to interact in their effects on *X*. These two assumptions are untestable.

### Mendelian randomisation methods

Two recent MR methods are Egger regression (ER) [15] and the weighted median estimator (WME) [9]. Unlike the two-stage least squares method, ER and WME are robust to violations of Condition *(c)*. ER is consistent regardless of the number of instruments that violate Condition (*c*), provided Conditions (*a*)-(*b*) and InSIDE hold, whilst WME makes an assumption on the proportion of instruments that satisfy (*c*) [9].

Assume linear additive dependencies between *Y* (the cardio-metabolite of interest), *U*, *X* (log BMI) and *Z* (the instrumental SNP of interest). Let $\hat {\beta }_{Y}$ and $\hat {\beta }_{X}$ denote the estimated slopes of the regressions of *Y* on *Z* and *X* on *Z*, respectively. Assume that the instruments are independent. Assume validity of Conditions (*a*)-(*b*) and InSIDE. Then the instrumental variable (IV) estimator of the causal effect of *X* on *Y* is $\hat {\beta }_{Y}/\hat {\beta }_{X}$. In a multi-instrument situation, each *j*th instrument contributes a separate IV estimate of the causal effect of *X* on *Y*, which we denote by $\hat {\beta }_{Yj}/\hat {\beta }_{Xj}$. When the IV estimates of several instruments, relative to a given outcome *Y*, show reasonable concordance, it would appear that a causal conclusion is defensible, pleiotropy notwithstanding. The ER method is based on the idea that concordance can be tested by regressing $\hat {\beta }_{Yj}$ on $\hat {\beta }_{Xj}$. A significant linear regression of the $\hat {\beta }_{Yj}$ on the $\hat {\beta }_{Xj}$ (with appropriate weights) will support (and provide an estimate of) the causal effect of *X* on *Y*. A point and interval estimate of the causal effect is obtained from the slope of the regression, whereas the mean pleiotropic effect is estimated by the intercept of the regression.

The WME method represents a different approach to the problem. Assume that Conditions (*a*)-(*b*) and InSIDE are valid, and that the instruments are independent, and at least half of them satisfy Condition (*c*). Then the median of the instrument-specific IV estimates will be a consistent estimate of the causal effect. The WME method implements this idea by introducing instrument-specific weights that take into account the uncertainty arising from the estimation of the *β*_*Yj*_. The WME estimator is consistent if at least half of the instruments satisfy Condition (c).

### Data analysis

In our study, the exposure *X* was defined to be the natural logarithm of the BMI. Analyses were performed conditional on age and sex. We performed a separate MR analysis for each of the 137 cardio-metabolites, with *Y* being defined in each analysis as the natural logarithm of the concentration of the studied metabolite. In each analysis the instrumental variables were selected from a genomewide sequence of SNPs on the basis of the statistical significance of the SNP’s association with the exposure [16]. Some of these SNPs were imputed. The final set of instruments consisted of SNPs with a call rate (both per SNP and subject) ≥ 0.95, minor allele frequency > 0.01, and absolute value of the Hardy-Weinberg equilibrium observed statistic < 4. These numerical thresholds have been frequently used in past genomewide genetic association studies. The causal effect of BMI on each of the 137 metabolites was estimated by using the ER and then the WME methods. Each of these two methods was used to calculate a point estimate, an interval estimate and a *p*-value for the causal effect of BMI on the metabolite in analysis.

We removed an extremely small number of outlying individuals in order to make the metabolite distributions approximately normal. We performed a hierarchical clustering of the log metabolite concentrations by using the Ward method, and by defining the distance between metabolites *j* and *k* to be given by $D_{jk} = \sqrt {1-R_{jk}^{2}}$, where *R*_*jk*_ is the sample Pearson correlation coefficient between the two log-concentrations.

## Results

The instrumental SNPs were selected from a genomewide list on the basis of their BMI associations, which we assessed by performing an age- and sex-adjusted regression of log BMI on each candidate SNP. The selection was based on commonly used criteria, that is, in such a way that their Wald test statistics were significant with *p*≤1×10^−5^, and the LD score between pairs of SNPs, as obtained by using the R snpStats package, never exceeded the threshold value of 0.05. This led to the selection of 18 SNPs, which we used as instrumental variables in our MR analysis. See Table 1 for information about the frequencies of the selected SNPs. The BMI-associations of the 18 instrumental SNPs did not necessarily remain significant at a 5% level after Benjamini-Hochberg adjustment for genomewide multiplicity.

**Table 1:** Percentage allele frequencies for the 18 instrumental SNPs in our DILGOM analysis, computed over 688 individuals

SNP label (dbSNP)	0	1	2
RS143298427	97.1	2.3	-
RS148966272	96.5	2.8	-
RS34364548	93.5	2	-
RS13413025	92.9	3.2	0.1
RS1502591	49.7	39.2	6.5
RS1504056	41.3	44.3	14.4
RS76755887	93.2	5.5	0.4
RS1109179	10.6	43.5	42.6
RS17052428	96.2	3.8	-
RS142181699	97.1	2	-
RS10269617	92.4	6.1	-
RS17159014	94	2.8	-
RS17109797	73.1	24.9	1.7
RS10459315	52.8	36.3	7.8
RS4782306	-	3.3	94
RS141336523	94.0	5.2	0.1
RS4816160	71.8	26.0	2.2
RS116920478	91.7	7.0	0.4

Allele coding: 0 stands for homozygous major, 1 for heterozygous, and 2 for homozygous minor, except for SNPs RS1109179 and RS4782306, where the homozygous categories are inverted

**Fig. 2:**
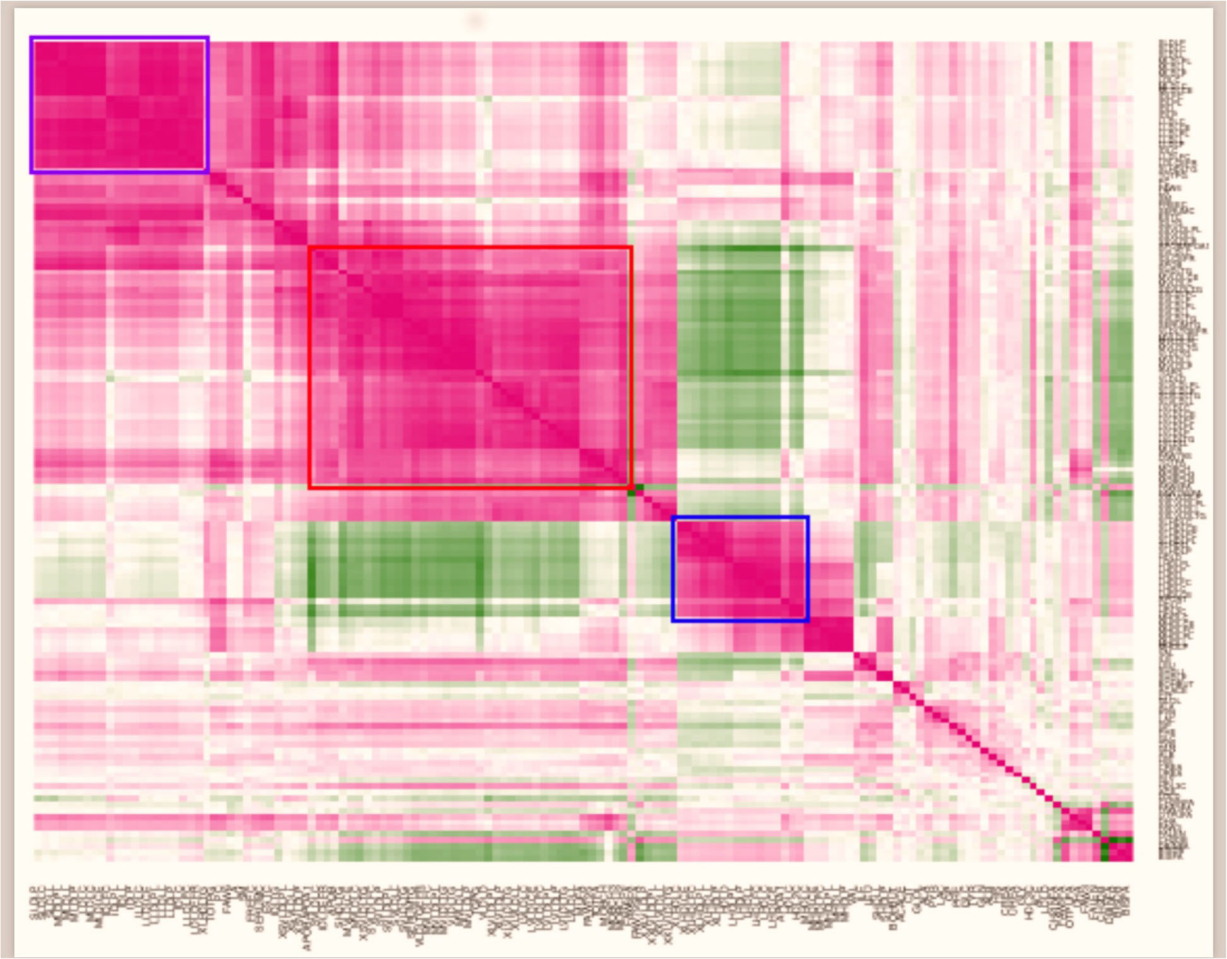
Heatmap representation of the Pearson correlations and clustering of the log-transformed metabolite concentrations

Figure 2 displays a heatmap based on the Pearson correlation coefficients between the 137 metabolites. The ordering of the metabolites along the axes is based on the results of a cluster analysis of their concentrations. Three main clusters are highlighted in the figure. They are delimited in the figure by a blue (top left), a red and a blue (in lower positions) enclosure, respectively. The top left cluster contains 22 metabolites, predominantly LDLs associated with the idea of “bad” (atherogenic) cholesterol and Intermediate Density Lipoproteins (IDLs). The red-bordered cluster contains further LDLs and VLDLs, these latter being characterised by extreme degrees of atherogenicity. The lower blue-bordered cluster contains HDL- related metabolites associated with a low degree of atherogenicity (“good” cholesterol). The “good” cholesterol concentrations turned out to be negatively correlated with those of the bad cholesterol metabolites in almost all the cases.

A finer subdivision of the main clusters, also based on the heatmap of Fig. 2, led to 10 clusters, formed by 22, 8, 7, 37, 5, 17, 6, 26, 4, 5 metabolites, respectively. Additional files 1 and 2 contain a description of the metabolite labels and a description of the metabolite memberships with respect to the finer clusters, respectively. Each of the 10 clusters contained a group of adjacent metabolites in the heatmap, the only exception being metabolite 75, which we included in cluster 6 on the basis of the pattern of its correlations. The ten clusters appeared as compact groups in a two-dimensional principal component representation of the set of metabolite log-concentrations, calculated on the basis of the metabolite-metabolite correlation matrix.

**Fig. 3:**
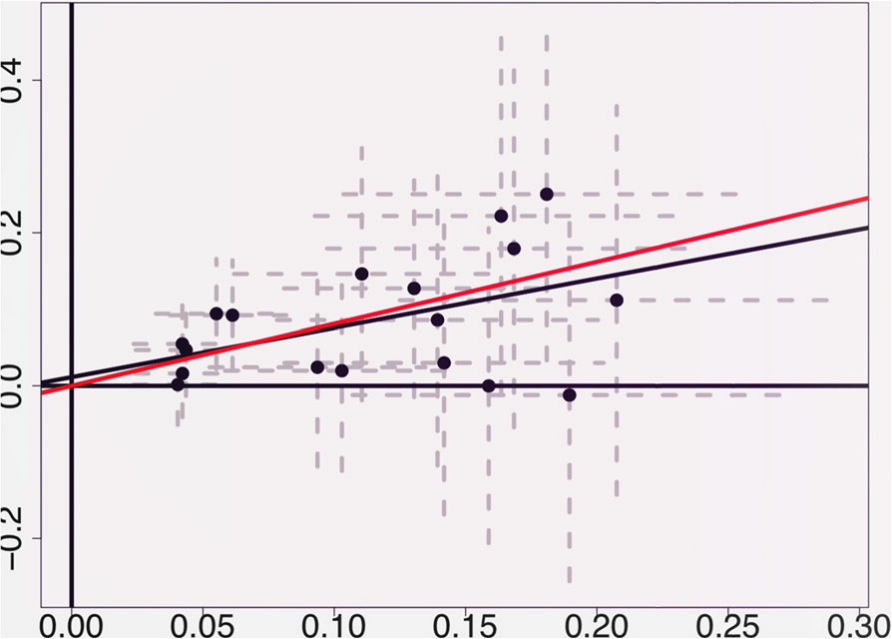
Illustration of Egger regression approach to Mendelian randomisation. Each point in the scatter plot represents an instrumental SNP. The vertical axis represents the estimated slope of the regression of *Y* (serum total triglycerides) on the SNP. The horizontal axis represents the estimated slope of the regression of *X* on the SNP. The black and red lines correspond to the Egger regression and weighted median estimate of the causal effect of *X* on *Y*, respectively. In this particular example, the intercept of the Egger regression is not significantly different from zero, which we interpret to indicate little evidence of directional pleiotropy (*p*=0.574). The estimates of the causal effect produced by Egger regression and by the weighted median estimator are both significant and in reasonable concordance. Grey dashed lines represent the 95 percent confidence intervals for the slopes in the regressions

Body mass, as measured in terms of BMI, was found to exert a significant causal effect on a large number of the studied metabolites. Figure 3 illustrates our analysis of the putative causal effect of BMI on one particular metabolite: the Serum Total Triglycerides (SERUMTG) concentration. The figure shows an Egger regression plot [9, 15], where each dot corresponds to one of the 18 instrumental SNPs. The ordinate of each SNP corresponds to the coefficient of that SNP in a linear regression of SERUMTG on that SNP, adjusted for age and sex. The abscissa of each SNP corresponds to the coefficient of that SNP in a sex and age-adjusted linear regression of log BMI on that SNP. The estimated slope of the regression through the points is represented in the plot as a black line. Under Conditions (*a*)−(*b*), and under InSIDE, this slope corresponds to the Egger estimate of the causal effect of log BMI on log SERUMTG. The slope of the red line corresponds to the WME [9] estimate of the same effect. The fact that the regression intercept is not significantly different from zero corresponds to lack of evidence of a non-null average pleiotropic effect. In such a case, the Egger regression estimate of the causal effect coincides with the Invariance Weighted Estimator [15].

Both the ER and the WME estimates of the causal effect of log BMI on log SERUMTG were significant (*p*=0.075 and 0.0053 respectively). The standard error of the WME estimates was calculated by using a bootstrap procedure, where the estimate of the causal effect is re-calculated from each of a long sequence of simulated datasets obtained from the original dataset via random selection of the sample individuals with replacement.

We also looked at the wider picture of the metabolite-specific causal effects. We found an interesting (if not unexpected) pattern. The causal effects of BMI on the metabolites in a given cluster tend to have the same sign, and this is the sign we expect on the basis of biological considerations: positive for highly atherogenic cholesterol metabolites, and negative for “good” cholesterol metabolites. These results suggest that an increase in BMI tends to increase the good cholesterol, and decrease the highly atherogenic cholesterol. This finding is further discussed in the next section.

We imputed the missing metabolite concentrations and SNPs with the aid of the R mice package. In the analysis of every new metabolite, we used the least absolute shrinkage and selection (LASSO) sisVIVE estimator [17] to estimate the number of instruments that violate Condition (*c*), so called “imperfect” instruments. We found this number to be always less than 9. In our WME calculations, the percentage of weight contributed by the imperfect instruments was always less than 50%, which ensures the consistency of the WME estimates of the causal effects.

**Fig. 4:**
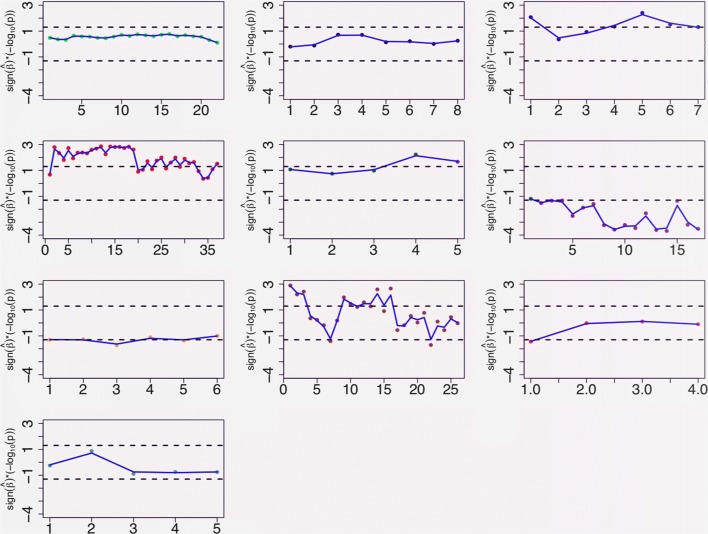
Scatter plot of the − log10*p*-values for the WME estimates of the causal effects of log BMI on each studied metabolite in each of ten clusters of metabolites. Each plot in the figure refers to the metabolites in a particular cluster, with cluster 1 represented by the left top subplot, and cluster 10 represented by the right bottom subplot. In the plots, the sign of each *p*-value equals the sign of the corresponding causal effect. The horizontal axes index the metabolites in each cluster, their order depending on the clustering method. The black dashed lines indicate thresholds for significance (± log100.05). The blue lines are smoothing splines

Figure 4 reports about the causal effects estimates obtained via WME, this method having been found to provide more precise estimates than ER in a simulation study performed by Bowden and colleagues [15]. In fact, in our study, WME estimates had more significant *p*-values than ER estimates. Figure 4 displays the signed *p*-values for the WME estimates, for each metabolite and cluster. These *p*-values were not adjusted for multiple testing, partly because there is little point in adjusting for multiple testing on a set of highly correlated metabolites [18]. Each subfigure corresponds to one of 10 fine clusters of metabolites. In each subplot, the horizontal axis indexes the set of metabolites contained in the corresponding cluster. Reported in each subplot are the −*log*_10_*p*values for the causal effect of log BMI on each metabolite in the relevant cluster, with the same sign as the sign of the causal effect estimate. The dotted horizontal lines indicate thresholds for significance of each individual estimate. Visual inspection of the figure reveals a tendency of the sign of the causal effect to be identical for each metabolite in each cluster. BMI appears to exert near-significant positive causal effects on almost all the LDL metabolites of cluster 1, which are known to be atherogenic. BMI appears to exert significant positive causal effects on most of the VLDL (very low density lipoprotein) metabolites of clusters 3 to 5, which are known to be extremely atherogenic, which is in accord with the available epidemiological evidence. All the estimates for the HDL metabolites that form clusters 6-7 are negative. In particular, our analysis provides convincing evidence that an increase in adiposity causes most of the HDL (high density lipoprotein, not atherogenic) metabolites to decrease in concentration.

Clusters 4, related to VLDL, and 6, to HDL, are significantly enriched in the number of significant causal estimates with log transformed BMI (*p*=2×10^−5^,0.000168; Benjamini and Hochberg *q*=0.00018,0.00084 respectively), whilst clusters 1 and 2, both related to LDL metabolites and the like, are significantly depleted in the same number (*p*<10^−6^, 0.00538; Benjamini and Hochberg *q*=2×10^−6^,0.02689 respectively), according to one-sided Fisher’s test for the enrichment or depletion of significant causal estimates and when assuming a hypergeometric random variable for the marginal counts in the 2×2 tables with the number of significant causal estimates in each cluster against the rest.

One referee correctly pointed out that the homogeneity of BMI causal effect within each cluster is partly a consequence of the clustering itself. However, we would emphasize that the metabolite clusters used in our analysis were strongly characterized from a functional and biological point of view. In particular, cluster 3 contains mildly atherogenic LDL-related metabolites, cluster 4 contains highly atherogenic VLDL-related metabolites, and cluster 6 contains HDL-related metabolites. The clustering was little or not sensitive to the clustering method used.

## Discussion

Würtz and colleagues [11] estimated the causal effect of BMI on each of a set of metabolic concentrations, via a two-stage least squares analysis based on instrumental information provided by a a set of SNPs associated with BMI. We carried out a similar analysis based on our DILGOM data, by using more robust MR methods, specifically methods that allow a subset of the instruments (which we are not asked to identify) to exert a pleiotropic influence on the outcome. Unsurprisingly, the results of our analysis agree with the notion that an increase in BMI tends to cause an increase in the concentrations of the atherogenic metabolites (such as those associated with low density cholesterol) and, perhaps more interestingly, a decrease in the concentrations of the non-atherogenic metabolites (such as those associated with high-density cholesterol).

Our genomewide search highlighted 77 SNPs very significantly associated with BMI, some of which were eliminated from the analysis at LD filtering stage. The set of these 77 SNPs did not include any of the 97 SNPs most significantly associated with BMI in a big meta-analysis on the European population [19]. Because our SNP data set contained 88 out of those 97 SNPs, this finding is surprising. Also, there was no overlap with the set of SNPs found in [20], nor with the set of 32 SNPs significantly related to BMI that form the genetic score in [11]. This finding may perhaps be ascribed to genetic differences between populations, and to BMI-related alleles being under-represented in our data set [20]. Another explanation of the finding could be the relative lack of power of our study, due to a smaller sample size than the previous studies, in conjunction with the small magnitude of the BMI-SNP associations [11, 19, 21].

Our estimates of the causal effects of BMI on the metabolites are in good accord with the available biological knowledge, and we regard them as a proof of principle in favour of using MR methods for dissecting the causal structure of disease.

## Conclusions

### Substantive

Due to the relatively small size of our sample, and to the cross-sectional nature of the study, our findings deserve future independent validation. We were pleased about their agreement with pre-existing biological knowledge. The results of our analysis of DILGOM data strengthen our confidence in the possibility to reduce the concentration of atherogenic VLDLs by reducing BMI via diet and physical exercise.

### Methodological

MR methods are rooted in the more general field of statistical causality [2]. As such, they are part of a framework that provides a new logic and language for reasoning about disease, and for developing a causal theory of illness on the basis of empirical data. We hope our DILGOM analysis provides a helpful proof-of-concept illustration of this fact.

Our careful review of the assumptions behind MR (many of which are, at best, only partially verifiable) should dispel any impression of MR representing an epidemiologist’s Holy Grail. Because of the untestable assumptions, we shall rarely be entirely convinced by the results of a single MR analysis. MR is a useful tool, but we must learn to use it in a wise and scientifically sensible way.

## Additional files

Additional file 1 Contains the metabolite labels in the same order as in Fig. 2. (DOCX 5 kb)

Additional file 2 Contains an Excel Table with the metabolite labels, in a cluster by cluster fashion. (XLSX 8 kb)

## Abbreviations

BMI: Body mass index
DILGOM: Diet lifestyle & genetic determinants of obesity & metabolic syndrome
ER: Egger regression (method for estimating a causal effect)
HDL: High density lipoprotein (“good” cholesterol)
IDL: Intermediate density lipoprotein
InSIDE: Instrument strength independent of direct effect
IV: Instrumental variable
LASSO: Least absolute shrinkage and selection operator
LD: Linkage disequilibrium
LDL: Low density lipoprotein (“bad” cholesterol)
MR: Mendelian randomisation
SERUMTG: Serum total triglycerides
SNP: Single nucleotide polymorphism
VLDL: Very low density lipoprotein
WME: Weighted median estimator (of a causal effect)
