# Supplementary material for: Mendelian randomisation analysis of clustered causal effects of body mass on cardiometabolic biomarkers
Source: BMC Bioinformatics. 2018 Jul 9;19(Suppl 7):195. doi: 10.1186/s12859-018-2178-2 (PMC6069804; doi:10.1186/s12859-018-2178-2)
Supplement: Supplementary file 1 — Contains the metabolite labels in the same order as in Fig. 2. (DOCX 5 kb) [file 12859_2018_2178_MOESM1_ESM.docx]

Supplementary_BMC_Bioinformatics.docx

Order of the metabolites (Inouye et al 2010) as in Figure 2:

[1] SLDLP SLDLC SLDLL MLDLPL MLDLL MLDLP LDLC MLDLC

[9] MLDLCE IDLFC IDLPL IDLL IDLP LLDLC LLDLCE LLDLPL

[17] LLDLL LLDLP IDLC LLDLFC LDLCEFR XLHDLTG TOTPG PC

[25] FAW6 LA SM FREEC SERUMC ESTC IDLTG XSVLDLPL

[33] XSVLDLL XSVLDLP APOBAPOA1 SVLDLC IDLCEFR APOB SHDLTG MVLDLCE

[41] MVLDLC XSVLDLTG SVLDLFC SVLDLP SVLDLPL SVLDLL SVLDLTG SERUMTG

[49] VLDLTGEFR MVLDLFC MVLDLPL MVLDLTG VLDLTG MVLDLL MVLDLP TGPG

[57] VLDLD XLVLDLPL XLVLDLP XLVLDLTG XLVLDLL LVLDLC LVLDLCE LVLDLFC

[65] LVLDLPL LVLDLP LVLDLTG LVLDLL MUFA FAW79S TOTFA MOBCH

[73] MOBCH3 MOBCH2 FAW6FA FAW79SFA XXLVLDLP XXLVLDLPL XXLVLDLL XXLVLDLTG

[81] XLHDLC XLHDLCE XLHDLFC XLHDLPL XLHDLL XLHDLP HDLD LHDLPL

[89] LHDLP LHDLL LHDLFC LHDLC LHDLCE APOA1 HDLC HDL2C

[97] MHDLC MHDLCE MHDLFC MHDLPL MHDLL MHDLP VAL ILE

[105] LEU SHDLL SHDLP BOHBUT ACACE CIT GLOL ALA

[113] PYR LAC GP PHE GLC TYR GLN ALB

[121] HIS CREA UREA GLY HDL3C ACE LDLD CH2INFA

[129] FAW3FA OTPUFA DHA FAW3 FALEN CH2DB DBINFA BISDB

[137] BISFA
